# Hyperthermia and targeting heat shock proteins: innovative approaches for neurodegenerative disorders and Long COVID

**DOI:** 10.3389/fnins.2025.1475376

**Published:** 2025-02-04

**Authors:** David M. Smadja, M. Marc Abreu

**Affiliations:** ^1^Paris Cité University, INSERM, Paris Cardiovascular Research Centre, Team Endotheliopathy and Hemostasis Disorders, Paris, France; ^2^Hematology Department, Hôpital Européen Georges Pompidou, Assistance Publique Hôpitaux de Paris-Centre Université Paris Cité (APHP-CUP), Paris, France; ^3^BTT Medical Institute, Aventura, FL, United States; ^4^BTT Engineering Department, Aventura, FL, United States

**Keywords:** hyperthermia, neurodegenerative disorders, serotonin, Long COVID, heat shock

## Abstract

Neurodegenerative diseases (NDs) and Long COVID represent critical and growing global health challenges, characterized by complex pathophysiological mechanisms including neuronal deterioration, protein misfolding, and persistent neuroinflammation. The emergence of innovative therapeutic approaches, such as whole-body hyperthermia (WBH), offers promising potential to modulate underlying pathophysiological mechanisms in NDs and related conditions like Long COVID. WBH, particularly in fever-range, enhances mitochondrial function, induces heat shock proteins (HSPs), and modulates neuroinflammation—benefits that pharmacological treatments often struggle to replicate. HSPs such as HSP70 and HSP90 play pivotal roles in protein folding, aggregation prevention, and cellular protection, directly targeting pathological processes seen in NDs like Alzheimer's, Parkinson's, and Huntington's disease. Preliminary findings also suggest WBH's potential to alleviate neurological symptoms in Long COVID, where persistent neuroinflammation and serotonin dysregulation are prominent. Despite the absence of robust clinical trials, the therapeutic implications of WBH extend to immune modulation and the restoration of disrupted physiological pathways. However, the dual nature of hyperthermia's effects—balancing pro-inflammatory and anti-inflammatory responses—emphasizes the need for dose-controlled applications and stringent patient monitoring to minimize risks in vulnerable populations. While WBH shows potential interest, significant challenges remain. These include individual variability in response, limited accessibility to advanced hyperthermia technologies, and the need for standardized clinical protocols. Future research must focus on targeted clinical trials, biomarker identification, and personalized treatment strategies to optimize WBH's efficacy in NDs and Long COVID. The integration of WBH into therapeutic paradigms could mark a transformative step in addressing these complex conditions.

## Introduction

Neurodegenerative diseases (NDs) are marked by the progressive deterioration of neuron structure and function, featuring distinctive molecular compositions of abnormal protein aggregates and irregular messenger RNA translation, which culminate in neuronal death (Storkebaum et al., 2023). Most of NDs lead to gradual deterioration of brain function and quality of life for millions of patients worldwide (Scheltens et al., 2021; Ben-Shlomo et al., 2024). As the population ages, the prevalence of NDs with a tendency to manifest later in life is expected to rise, affecting many seniors. NDs, despite their varied causes, share strikingly similar cellular and molecular pathways (Meriin and Sherman, 2005; Storkebaum et al., 2023). This similarity holds promise that discoveries and treatments developed for one ND could be applied to combat others. Common NDs, such as Parkinson diseases (PDs) or Alzheimer's disease (ADs) and certain inherited conditions linked to polyglutamine (polyQ) expansions like Huntington's Disease (HDs) and various spinocerebellar ataxias, share a pathology characterized by the accumulation and deposition of abnormal polypeptides/amyloid product able to be formed in different cellular locations or even outside the cell (Wetzel, 2002; Thirumalai et al., 2003). A key feature of amyloids is their extensive beta-sheet structures, stabilized by numerous hydrogen bonds, leading to the formation of amyloid fibrils. These fibrils are structurally so similar that antibodies developed against one type of amyloid peptide can recognize fibrils from a wide range of unrelated peptides, such as polyQ, but not their soluble forms (O'Nuallain and Wetzel, 2002; Kayed et al., 2003; Kozin et al., 2023). Normally, cells manage the folding of polypeptides into their functional conformations soon after synthesis or transport to the right cellular compartment. This process is crucial as the buildup of misfolded, non-functional polypeptides can be toxic. Evolution has thus equipped cells with defense mechanisms like molecular chaperones and the ubiquitin-proteasome degradation system (UPS) to handle and mitigate the accumulation of unfolded proteins caused by mutations, synthesis errors, or inefficient folding (Ataei et al., 2024; Kassab et al., 2024). Chaperones play a dual role in helping the correct folding of polypeptides and shielding cellular proteins from damage due to stressors like heat while also assisting in the refolding of denatured proteins (Frydman, 2001; Goldberg, 2003; Chen and Johansson, 2024). Hyperthermia (HT), also known as thermal therapy, refers to the intentional elevation of tissue temperature to ~39–42°C, typically maintained for about 1 h (Mallory et al., 2016; Smadja, 2024). This treatment modality has been widely investigated, especially in oncology, where it is often used alongside radiotherapy, chemotherapy, and immunotherapy to enhance therapeutic outcomes. Additionally, HT has gained attention as a promising intervention for depression, offering an alternative or adjunctive treatment to support mood regulation (Janssen et al., 2016). It is essential to distinguish therapeutic HT from two other temperature-related medical conditions or procedures: first, malignant HT is a potentially life-threatening reaction to certain medications (Kollmann-Camaiora et al., 2017); second, thermal ablation involves heating tissues to temperatures exceeding 44°C, typically with the goal of destroying malignant cells or problematic tissues (Wu, 2016). These conditions differ significantly from therapeutic HT in both purpose and temperature range. HT therapy can be further categorized based on specific parameters that influence its application and effectiveness. The depth of heating distinguishes between superficial, deep, and interstitial HT. Superficial HT targets tissues close to the body surface (Dobšíček Trefná et al., 2017), while deep HT reaches tissues or organs located deeper within the body (Bruggmoser et al., 2011). Interstitial HT, on the other hand, involves inserting heating elements directly into tissues, allowing precise, localized temperature control (Dobšíček Trefná et al., 2019). The volume of heating is another important parameter, as HT may be applied locally to small, targeted areas; in a loco-regional manner to treat larger areas or regions, which may include nearby tissues or an entire organ; or as whole-body HT (WBH), where the temperature of the entire body is raised, typically to produce systemic effects (Heckel-Reusser, 2022). Lastly, HT is defined by the range of temperature used. Moderate HT involves controlled, moderate heating, whereas mild HT produces a gentler temperature elevation. Fever-range HT mimics natural fever temperatures, which can stimulate immune responses and may be particularly beneficial in certain therapeutic contexts. The application of HT in the realm of neurodegeneration seeks to utilize thermal stress as a means to either curb the degenerative processes or facilitate the recovery of neuronal functions. A landmark moment in the history of HT as a medical intervention was when Dr. Julius Wagner-Jauregg was awarded the Nobel Prize in Medicine in 1927 for his innovative treatment of dementia paralytica by inducing high fever through malaria infection (Gartlehner and Stepper, 2012; Daey Ouwens et al., 2017). Dementia paralytica, also once termed general paresis of the insane, represents a critical stage of syphilis infection characterized by profound cognitive decline and physical deterioration. Dementia paralytica shares similarities with NDs, including significant cognitive and motor dysfunctions, thereby highlighting the Nobel Prize-recognized role of HT in opening new therapeutic avenues for NDs (Dewhirst et al., 2005; Mallory et al., 2016). This method underscored the potential therapeutic benefits of induced HT, setting a precedent for its exploration in the treatment of NDs.

This article aims to investigate the potential impact of HT on NDs by delving into its operational mechanisms, the supporting research evidence, potential advantages, obstacles, and the future prospects of this novel therapeutic avenue.

## HSPs and NDs

**Figure 1:**
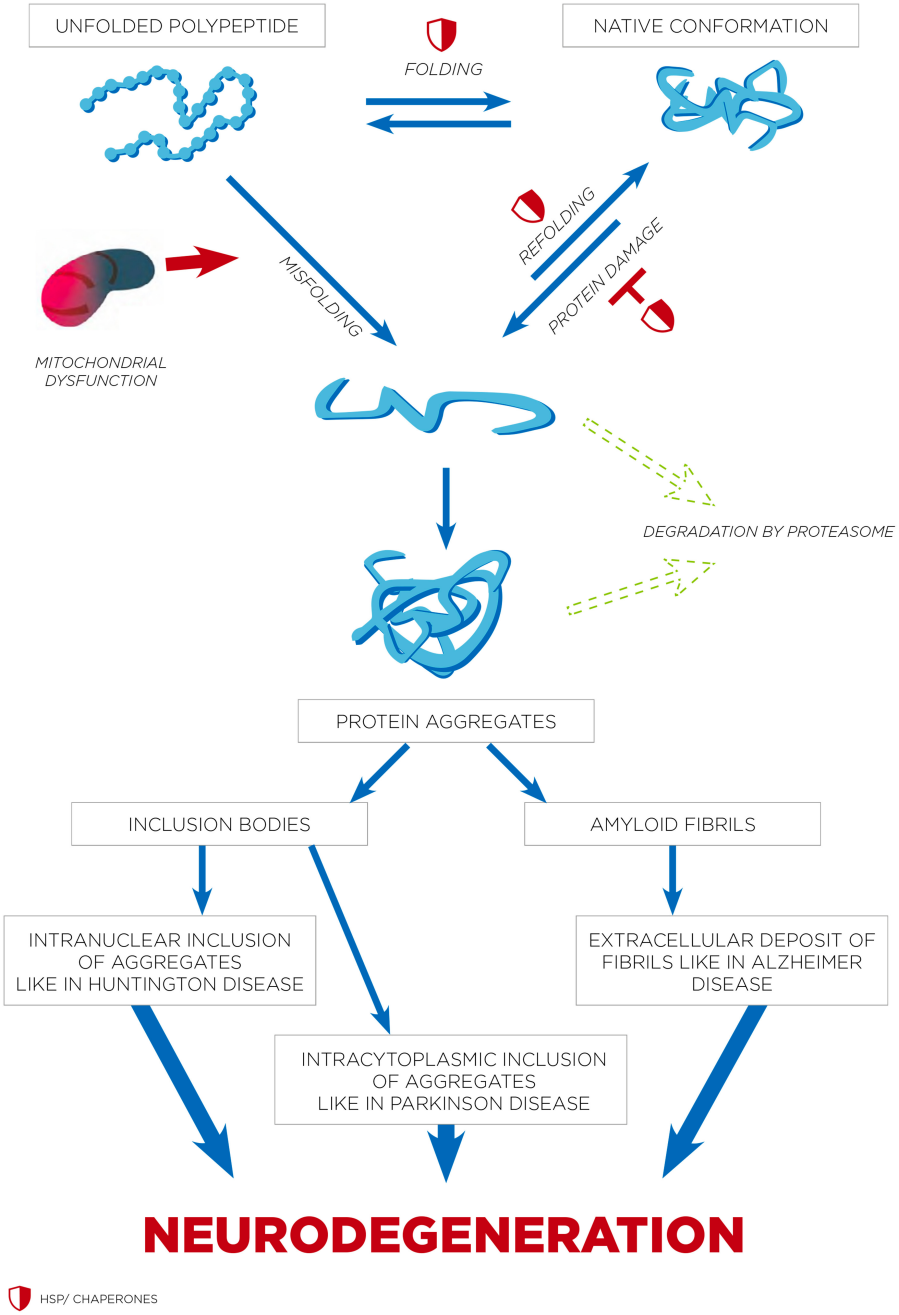
HSPs and inhibition of apoptosis. In response to stress, HSF-1 becomes activated. This activated HSF-1 then promotes the expression of HSPs genes. As a result, HSPs such as HSP70 and HSP90 are produced. These HSPs play a critical role in inhibiting the release of cytochrome c from the mitochondria. The inhibition of cytochrome c release is crucial because cytochrome c is necessary for the activation of APAF-1 (Apoptotic Protease Activating Factor-1). Without cytochrome c, APAF-1 cannot activate, thereby preventing the apoptotic pathway from proceeding. In addition to inhibiting cytochrome c release, HSPs also inhibit pro-apoptotic factors like Bax and Bak, while stabilizing anti-apoptotic factors such as Bcl-2. Furthermore, HSPs inhibit the activation of caspases, which are enzymes critical for the execution of apoptosis. The combined effect of these actions by HSPs—preventing cytochrome c release, inhibiting APAF-1 and caspase activation, and modulating pro- and anti-apoptotic factors—ultimately leads to the inhibition of apoptosis.

**Figure 2:**
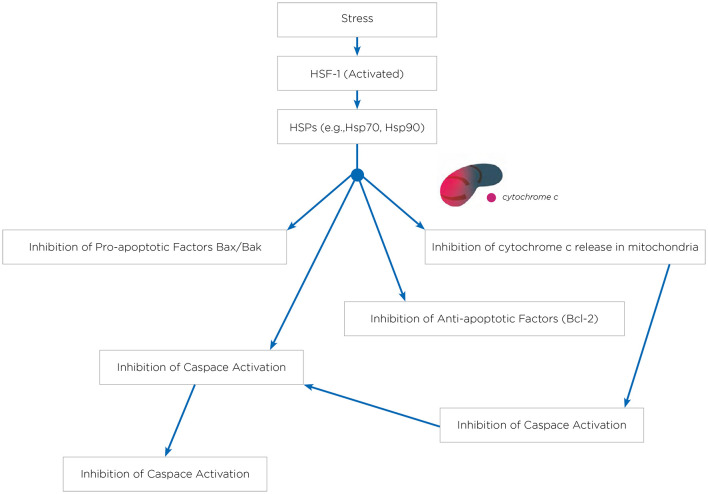
HSPs and refolding of protein in neurodegenerative disorders. The process of protein folding involves transitioning through intermediate stages that are stabilized by molecular chaperones such as HSPs. Different types of protein aggregates are seen in various neurodegenerative disorders. Both intranuclear and cytoplasmic inclusions are associated with conditions like HDs and PDs. While amyloid fibrils are usually found in the extracellular space, there have been instances of intracellular amyloid fibrils as well.

Hyperthermia engages multiple biological mechanisms that may positively influence the pathophysiology of NDs. HT triggers a range of beneficial cellular responses, one of which includes the induction of HSPs (Table 1; Sharma and Hoopes, 2003; Lukácsi et al., 2024). These proteins are vital for protein folding, repair, and degradation. The HSR, a natural propensity of most HSPs, helps cells adapt to stress by preventing protein aggregation, blocking apoptosis, and enhancing cell survival. Both environmental and physical stressors can activate these protective responses of HSPs, which are integral to maintaining cellular functions (Singh et al., 2024). One of the primary protective actions of HSPs is the inhibition of apoptosis (Ikwegbue et al., 2017). The heat shock transcription factor 1 (HSF-1), a crucial regulator of this process, modulates apoptosis pathways. Disruption of HSF-1 can led to apoptosis and cell death. HSPs inhibit apoptosis beginning at the critical juncture where mitochondria release cytochrome c (Figure 1). By operating at various stages of the apoptotic pathways, HSPs provide cellular protection by neutralizing numerous targets involved in apoptosome formation (Beere et al., 2000). HSPs obstruct the factors that promote cytochrome c secretion, and further inhibit the binding of cytochrome c to the apoptotic protease activating factor-1 (Apaf-1; Beere et al., 2000). The interaction between Apaf-1 and HSP70 is particularly noteworthy, as it plays a crucial role in hindering mitochondrial apoptotic activity (Ko et al., 2015; Wei et al., 2024). Meanwhile, HSP27 directly prevents the release of cytochrome c (Bruey et al., 2000). Consequently, the induction of HSPs serves as an effective mechanism for cell survival and cytoprotection, both of which are central to the pathology of NDs. HSPs are also and especially of chaperones protein that are essential components in cellular biology (Singh et al., 2024), primarily functioning to ensure proper protein folding and preventing the aggregation of misfolded proteins, which could potentially lead to cellular dysfunction and NDs (Meriin and Sherman, 2005). Chaperones assist in the correct folding of newly synthesized proteins (Saibil, 2013). They provide a secluded environment where a protein can fold without the risk of aggregating with other proteins. This is crucial because the correct 3D shape of a protein is essential for its function. If proteins begin to unfold or misfold due to cellular stress or mutations, chaperones can bind to these misfolded proteins and help them refold into their correct shapes. Chaperones can recognize exposed hydrophobic patches on the surface of unfolding or misfolded proteins, which are prone to aggregation (Marino Gammazza et al., 2016; Macario and de Macario, 2020; Scalia et al., 2021). By binding to these patches, chaperones prevent the proteins from sticking together, thus inhibiting aggregation. HSPs are a subset of chaperones that are highly expressed in response to HT (Gao et al., 2016) but also to some toxins or oxidative stress. This response helps protect cells from damage by ensuring proteins maintain their functional conformations. Due to their role in protein folding and stress response, HSPs are implicated in NDs (Meriin and Sherman, 2005). In NDs, where protein misfolding and aggregation are common pathological features, HSPs can help mitigate these issues, potentially slowing disease progression (Figure 2). Conditions like ADs, PDs, and amyotrophic lateral sclerosis (ALS) are known as “protein misfolding diseases” (Table 2), marked by the buildup of incorrectly folded proteins prone to aggregation within neurons (Figure 2). Moreover, Creutzfeldt-Jakob disease is a rare and fatal NDs caused by misfolded prion proteins that induce abnormal aggregation of normal cellular prion proteins (PrP), leading to the formation of toxic protein aggregates (Müller et al., 2000; Kamps et al., 2024; Table 2). HSPs belong to a group of polypeptides whose production increases during stress periods to help cells preserve their capacity to oversee protein balance (Fink, 1999; Saibil, 2013). This encompasses the refolding of proteins that have misfolded and/or clumped together through processes powered by adenosine 5′-triphosphate (ATP) and directing proteins that cannot be repaired toward breakdown via the ubiquitin-mediated proteolytic pathway (Fink, 1999; Saibil, 2013). In situations of disease, the quality control mechanisms for proteins may lead to the buildup of improperly folded proteins, stemming from mutations or excessive volumes of target proteins. HSPs are categorized into several groups according to their size: large HSPs that depend on ATP for their chaperone activity [including 90 kDa (HSP90), 70 kDa (HSP70), 60 kDa (HSP60)] and small HSPs (ranging from 15 to 30 kDa), notably HSP27, which function without ATP. HSP90 and HSP70 are among the most studied and thus most well-understood members of the HSPs family (Fink, 1999; Saibil, 2013). HSPs are crucial to the heat shock response (HSR) pathway, overseen by HSF-1, the principal transcription factor for various HSPs. This pathway activates in response to not just thermal stress, but also other stressors like low oxygen levels and exposure to pollutants. Following its activation and movement into the cell nucleus, HSF-1 attaches to DNA, starting the transcription of mRNAs that instruct the synthesis of new HSP90, HSP70, and HSP27 proteins to manage stressed proteins. Any deficiencies within the Chaperone System are known to cause disorders termed chaperonopathies (Macario and de Macario, 2020), which are essentially disorders arising from structural or functional anomalies in chaperones, whether through genetic mutations or acquired issues, leading to chaperone dysfunction. An important subset of these disorders, neurochaperonopathies, includes conditions such as NDs and neuromuscular diseases (Marino Gammazza et al., 2016; Scalia et al., 2021). The disruption of protein homeostasis is marked by the emergence and accumulation of abnormal protein aggregates, such as α-synuclein in PDs (Chinchilla et al., 2024), huntingtin in HDs, and β-amyloid plaques in ADs (Table 2). These disturbances manifest as the formation of misfolded proteins, disrupting normal biological pathways and preventing proteins from fulfilling their intended roles. Consequently, this leads to the proliferation of neurological conditions characterized by these misfolded proteins. Genetic predispositions may play a crucial role, although no single mutation has been pinpointed as a direct cause, comparisons between familial (early-onset) and sporadic (late-onset) instances suggest a genetic basis for early and rapidly progressing forms of ADs. Furthermore, chaperones are highly sensitive to environmental conditions and biochemical changes, leading to acquired neurochaperonopathies over time (Scalia et al., 2021). Alterations in chaperone proteins like HSP70 and HSP90 have been linked to ADs (Campanella et al., 2018). Tau proteins and β-amyloid plaques are two hallmark features associated with ADs and are both regulated by chaperones/HSPs. Indeed, both tau proteins and β-amyloid plaques are crucial in understanding the pathology of AD, but they operate through distinct mechanisms within the brain (de Paula et al., 2009). Tau is a microtubule-associated protein found predominantly in neurons' axons, where it plays a critical role in stabilizing microtubules and supporting neuronal structure and function (Mietelska-Porowska et al., 2014). In AD, abnormal hyperphosphorylation of tau proteins occurs, leading to their detachment from microtubules. These detached tau proteins then aggregate to form neurofibrillary tangles (NFTs) inside neurons. The accumulation of NFTs disrupts neuronal communication and contributes to cell death (Mietelska-Porowska et al., 2014). β-Amyloid (Aβ) plaques are extracellular deposits found in the brains of individuals with AD. They are primarily composed of amyloid-beta peptide, a fragment derived from the amyloid precursor protein (APP; Sighencea et al., 2024; Zhou et al., 2024). APP is a transmembrane protein that, when abnormally processed by enzymes β- and γ-secretase, yields amyloid-beta. This peptide can aggregate into oligomers and fibrils, eventually forming plaques that disrupt cell-to-cell signaling and facilitate neuroinflammation, contributing to neuronal damage. While tau proteins and β-amyloid plaques are separate entities, a complex interplay between them exist in the progression of ADs (Sighencea et al., 2024; Zhou et al., 2024). One prevailing hypothesis is that the accumulation of β-amyloid plaques may precede and potentially instigate the pathological aggregation of tau, leading to a cascade of neurodegenerative processes (Garbuz et al., 2021). β-Amyloid oligomers are thought to induce tau hyperphosphorylation and misfolding, which in turn accelerates tau aggregation and tangle formation (Campanella et al., 2018). This interaction suggests that β-amyloid acts as a trigger for tau pathology, which then drives the neurodegenerative process forward, leading to the cognitive decline observed in AD (Sighencea et al., 2024; Zhou et al., 2024). HSPs and the HSR predominantly serve as protective agents with the capability to mitigate AD manifestations. HSPs are known for their ability to dismantle amyloid aggregates and halt further accumulation by blocking the foundational and growth phases of amyloid fibrils' formation. However, AD may be characterized by a modified HSP expression, as the HSR frequently becomes imbalanced in individuals who are aged or overweight—conditions commonly associated with AD (Campanella et al., 2018). The activation of HSF1 is impeded, reducing its nuclear migration and subsequently diminishing HSP gene activity. Among these, HSP90 stands out as the most prevalently found HSPs within eukaryotic organisms (Jackson, 2012). It primarily resides within the cytoplasm, engaging in anti-inflammatory actions and rectifying aberrant proteins. HSP90 plays a key role in preventing amyloid build-up and the formation of Aβ peptides. Notably, HSP90 outside the cell activates phagocytes and initiates the Toll-like receptor-4 pathway (Fan et al., 2022), leading to the breakdown of Aβ peptides (Kakimura et al., 2002). HSP90 counters Aβ-induced harm by attaching to improperly folded Aβ peptides, either preventing their further accumulation through an ATP-independent route or altering the Aβ structure to a less aggregative form via an ATP-reliant method. Additionally, HSP90 influences the metabolism of the tau protein (Batko et al., 2024). However, in scenarios where tau protein accumulates, the function of HSP90 contributes to its further aggregation. HSP90 chaperones connect with hyperphosphorylated tau, triggering their breakdown. HSP90's functionality spans broadly, hence, post-translational adjustments and the involvement of co-chaperones critically influence HSP90 activity. This encompasses HSP90's acetylation/phosphorylation and its integration into more complex formations with other HSPs, particularly HSP70 and HSP40. The collective action of the HSP90/70/40 complex can decelerate Aβ pileup, contingent on the chaperone concentration (Batko et al., 2024). Moreover, this complex's presence or activity tends to inversely match tau accumulation within brain specimens from AD model mice and human AD patients, highlighting its protective capacity. The HSP70 family encompasses a diverse set of chaperones scattered across various cellular locales, united by a commonality in molecular mass and the presence of a substrate-binding domain at their C-terminal for polypeptide interaction, alongside an N-terminal nucleotide-binding domain (NBD) that facilitates ATP hydrolysis. Within the context of AD, HSP70 proteins exhibit safeguarding functions through several pathways, such as inhibiting Aβ oligomer formation (Evans et al., 2006), transitioning Aβ into a structure less prone to amyloidogenesis, enhancing the breakdown of Aβ by specific enzymes, and reestablishing tau equilibrium by promoting the clearance of phosphorylated tau aggregates, likely via the ubiquitin-proteasome and/or autophagy pathways (Moyano et al., 2021; Batko et al., 2024). The synergy between HSPs, notably between HSP70 and HSP90, plays a crucial role in disassembling tau proteins and averting tau aggregation, thereby positioning them as compelling candidates for therapeutic intervention (Moll et al., 2022). This stems from their direct involvement in tau-related pathology and the extensive therapeutic avenues opened through modulating these chaperones' interactions, such as the observed neuroprotection against β-amyloid toxicity linked to elevated HSP70 levels in AD research (Magrané et al., 2004). Similarly, in a model of PDs, enhanced expression of HSP70 was shown to mitigate the harmful effects of α-synuclein on dopaminergic neurons (Auluck et al., 2005). This response is regulated at the genetic expression level by HSF1, prompting exploration into HSF1's role in managing HSPs and examining compounds that could modulate HSF1, aiming to address NDs linked to misfolded proteins (Hekmatimoghaddam et al., 2017). HSP70 protein has been described to have higher concentrations within the nervous system of mammals than in non-neuronal tissues, with a particularly high presence within the neuron cell bodies (Loones et al., 2000). Recent investigations have compared the abundance of HSP70 across various neuron types that are selectively vulnerable in distinct NDs (Lyon and Milligan, 2019). Specifically, motor neurons in the spinal cord, targeted by less common conditions like ALS, show significantly elevated HSP70 levels (Lackie et al., 2017). In contrast, neurons located in the hippocampus and entorhinal cortex, which are compromised in more prevalent conditions such as ADs, exhibit lower HSP70 concentrations (Sabirzhanov et al., 2012). Neurons in the substantia nigra, affected by diseases of intermediate prevalence like PDs, display moderate HSP70 levels (Planas et al., 1997). Thus, the differences in HSP70 levels among neuron types may influence their capacity to shield against protein misfolding disorders, which seems to align with the varying incidences of these diseases within the human population (Yenari, 2002). This suggests that neurons might depend on their inherent HSP70 levels as an initial protective mechanism against the protein misfolding and aggregation triggered by stress or related to NDs (Zatsepina et al., 2021).

**Table 1:** Main heat shock proteins, their location, and cellular function.

**HSP**	**Location**	**Cellular function**
HSP 110 (Somu et al., 2024)	Cytosol and nucleus	Protein disaggregation, thermotolerance, Helps immune response and complexes with HSP70 to promote protein refolding and cell survival under stress
HSP 90 (Whitesell and Lindquist, 2005)	Cytoplasm	Stabilization of misfolded proteins, refolding, and degradation. It also facilitates signal transduction and important roles in cancer and sarcomere formation as well as in myosin folding
HSP 70 (Mayer and Bukau, 2005)	Cytoplasm and nucleus	Aids protein assembling, folding, and transport. Also participate to degradation of improperly folded peptides and translocation of organelles
HSP 60 (Malik and Lone, 2021)	Cytoplasm and mitochondria	Assists in protein folding, prevents protein aggregation, and assembling of unfolding proteins via the formation of the hetero-oligomeric complex
HSP 47 (Thienel et al., 2023)	Endoplasmic reticulum	Collagen-specific molecular chaperone. Its major role is to assist in the proper folding and assembly of procollagen molecules within the endoplasmic reticulum. Involvement in platelet biology and thrombosis.
HSP 27 (Zou et al., 2023)	Cytosol, endoplasmic reticulum, and nucleus	Facilitates refolding of denatured proteins (chaperoning activity) and serves as a biomarker in many cellular diseases such as cancer

**Table 2:** Main NDs due to protein misfolding and aggregate formation.

**Diseases**	**Proteins involved**
Alzheimer's disease (AD)	Aβ and tau
Parkinson's disease (PD)	α-Synuclein and tau
Huntington disease (HD)	Huntingtin
Prion	PrP
Tauopathies	Tau
Lewy bodies dementia	α-Synuclein and ubiquitin

## Pharmacological strategies for modulating HSPs in NDs

Exploring chaperone modification or HSPs manipulation in NDs like ADs, PDs, and Amyotrophic Lateral Sclerosis (ALS) represents a dynamic field of study, albeit still in the exploratory phases (Sarah Kishinevsky and Wenjie Lou, 2013). HSPs offer promising therapeutic targets due to their crucial roles in protein folding, refolding, and degrading misfolded proteins under stress, which are common issues in these disorders. Pharmacological strategies are under investigation, focusing on enhancing HSPs expression or modifying their function. Examples of substances targeting HSPs are detailed in Table 3. Notably, activating HSF1, a key regulator of HSPs expression, is a significant area of research (Liu et al., 2023). Arimoclomol is a promising molecule in this regard, especially for ALS, where it has shown potential in clinical trials to slow disease progression by increasing HSPs expression (Kalmar and Greensmith, 2017). This molecule is particularly noteworthy for its ability to extend survival and improve motor function in ALS models by enhancing HSF1 activation and increasing levels of HSP70 and HSP90 in motor neurons, even when treatment is initiated after symptom onset (Batulan et al., 2006). Furthermore, studies on recombinant human HSP70 (rhHSP70) have indicated its potential in experimental models of Niemann-Pick disease type C (NPC), a condition characterized by reduced myelination and cerebellar atrophy (Sweeney et al., 2017). Treatments with bimoclomol or rhHSP70 in NPC models have shown significant improvements in myelination and an increase in mature oligodendrocytes, alongside enhancing phosphorylated Fyn kinase activity (Nánási and Jednákovits, 2001; Kalmar and Greensmith, 2017). HSP60 modulators, including epolactaene and ETB (Epolactaene Analog 3; Sun et al., 2012; Meng et al., 2018), are compounds originally derived from marine fungi, such as *Penicillium* species, that specifically target HSP60, a mitochondrial chaperone crucial for protein folding and cellular protection within the mitochondria. Epolactaene binds irreversibly to HSP60, a mechanism linked to its antitumor effects (Sun et al., 2012; Meng et al., 2018). This interaction selectively disrupts mitochondrial function in cancer cells, promoting apoptosis and reducing cell viability. ETB, a structural analog of epolactaene, was developed to enhance anticancer activity. It retains similar binding properties to HSP60, making it a promising candidate for cancer therapy by targeting mitochondria in a comparable manner, impairing energy production and inducing programmed cell death in cancer cells (Meng et al., 2018). This highlights HSP70's role in promoting myelination and its therapeutic promise for NDs (Scalia et al., 2021). Additionally, the role of HSP70 in preventing Aβ accumulation and tau protein breakdown has been acknowledged, with research into rhodacyanine-based compounds and phenothiazines like Methylene Blue and Azure C, which inhibit HSP70's ATPase activity, leading to a reduction in tau levels and prevention of tau aggregation (Batko et al., 2024). The development of HSP90 inhibitors offers another promising avenue, as these substances can indirectly boost the expression of other stress response proteins like HSP70 by lifting feedback inhibition on HSF1 (Lyon and Milligan, 2019). These inhibitors, including geldanamycin and its derivatives as well as purine scaffold inhibitors and C-terminal inhibitors like celastrol and novobiocin, are being studied for their capacity to mitigate the impact of protein misfolding disorders (Sinnige et al., 2020; Batko et al., 2024). Celastrol, derived from traditional Chinese medicine, is noteworthy for its potential neuroprotective effects in NDs, attributed to its ability to induce HSP70 expression through HSF1 activation (Kakkar et al., 2014). This compound, in combination with other HSP90 inhibitors, possesses anti-inflammatory and immunosuppressive properties, and offers protection against β-amyloid toxicity. Its effectiveness in animal models of NDs further underscores the therapeutic potential of HSPs modulators in combating these conditions (Ansar et al., 2007; Batko et al., 2024).

**Table 3:** Pharmacological modulation of heat shock proteins (HSPs): targets, drugs, and therapeutic effects.

**Target**	**Drug**	**Effect**
HSF-1	Arimoclomol (derivative of hydroxylamine)	Activates HSF1, upregulating HSP expression—induces a protective stress response and neuroprotection
	Bimoclomol (synthetic small molecule that is also a derivative of hydroxylamine)	
HSP 60	Epolactaene (naturally occurring compound originally isolated from a marine fungus known as *Penicillium* sp.) or ETB (epolactaene analog 3)	Interaction disrupts the function of Hsp60; neuroprotective and anticancer activities
HSP 70	Rhodacyanine-based compounds (MKT-077; YM-01; YM-08)	MKT-077: This compound is a mitochondrial-targeted inhibitor of HSP70 and selectively induces apoptosis in cancer cells. Its mechanism involves destabilizing mitochondrial function, which leads to cell death in cells that rely heavily on HSP70 for survival, such as many cancer types. YM-01 and YM-08: inhibit HSP70 by impairing its ATPase activity, thus disrupting protein folding. They have shown promise in studies targeting tumor cells.
HSP 70	Phenothiazines (Methylene Blue and Azure C)	Neuroprotective and anti-aggregation effects
HSP 90—N-terminal inhibitor	Geldanamycin (Derived from the bacterium *Streptomyces hygroscopicus*)	Inhibition of tau protein—disrupts HSP90's role in stabilizing oncogenic proteins by inhibiting its ATPase function, causing proteasomal degradation of HSP90 client proteins
HSP 90—C-terminal inhibitor	Celastrol (quinone methide triterpene derived from traditional Chinese medicine, specifically from the root of *Tripterygium wilfordii*)	Mitigate the impact of protein misfolding disorders—neuroprotection from AB toxicity
HSP 90—C-terminal inhibitor	Novobiocin (aminocoumarin antibiotic)	

## HT modulates HSPs: Insights for NDs pathophysiology and treatment

Hyperthermia, or elevated body temperature, significantly impacts the expression and function of HSPs, which are a critical component of the cell's response to stress (Moseley, 1997; Sharma and Hoopes, 2003). The relationship between HT and HSPs is a key area of interest in both research and clinical settings for several reasons: HT triggers the HSR leading to the upregulation of HSPs. HSPs like HSP70, HSP90, and small HSPs become highly expressed under conditions of elevated temperatures. Their role is to protect cells from the detrimental effects of stress and heat, including protein denaturation and aggregation, by assisting in the proper folding and refolding of proteins. By facilitating protein repair and preventing aggregation, HSPs play a crucial role in cell protection during HT. This protection is crucial in tissues sensitive to temperature changes, such as neurons in the brain (Horowitz and Robinson, 2007). The HSR, including the expression of HSPs, has therapeutic implications. Thus, HT may play a role in protecting against NDs characterized by protein misfolding and aggregation, such as AD and PDs (Zulkifli et al., 2003). Enhancing the expression of HSPs could potentially help in stabilizing misfolded proteins and preventing their aggregation, a hallmark of many NDs. Repeated exposure to mild HT can lead to the development of thermotolerance, where cells exhibit increased resistance to subsequent heat stress (Amorim et al., 2015). This adaptation is largely mediated by the increased expression of HSPs, which enhance the cellular protective mechanisms. HSPs are notably significant for their involvement in enhancing thermo-tolerance (Parsell et al., 1993). The discovery that HSPs can be induced in living animals was made after their core body temperatures were raised to between 42.0 and 42.5°C for 15 min (Currie and White, 1981, 1983). Specifically, HSP70 levels rise in various neural tissues, such as the retina, cerebral hemisphere, cerebellum, and brainstem, starting as early as 1.5 h after exposure to increased temperatures and can remain elevated for up to 24 h (Manzerra et al., 1997; Krueger et al., 1999). In rats, this spike in HSP70 within the forebrain and cerebellum is notably concentrated around synapses, including both pre- and post-synaptic structures, which suggests HSP70 may have a critical role in the protection and repair of synaptic proteins after HT (Bechtold et al., 2000). Similarly, the expression of HSP27 is triggered in the rat's forebrain and hippocampus following heat exposure (Krueger-Naug et al., 2000; Bechtold and Brown, 2003). A day after experiencing HT, there's a noticeable increase in HSP27 levels across several brain regions—the cerebral cortex, hippocampus, cerebellum, and brainstem—within both neuroglia and neurons, and specifically within certain neuronal groups in the hypothalamus (Krueger-Naug et al., 2000). Intriguingly, in the spinal cord, HT leads to a rise in HSP70 levels in glial cells but not in neurons, observable 4 h post-treatment (Sasara et al., 2004). Tu et al. highlighted the changes in HSP70 and HSP90 levels in gastric tumors following transient hyperthermic intra-peritoneal chemoperfusion (HIPEC) therapy (Tu et al., 2018). Their research indicated that performing a second HIPEC session 24 h after the first could reduce the resistance to chemotherapy and heat treatment triggered by increased serum HSP70/90 levels from the initial HIPEC session. Shetake's group previously demonstrated the function of HSP90 in enhancing sensitivity to radiation and heat in a mouse fibrosarcoma tumor model treated with magnetic HT (MHT; Shetake et al., 2015; Tu et al., 2018) and also its relevance as predicting factor for response to HT (Shetake et al., 2023). HT can also enhance the process of autophagy, where cells degrade and recycle cellular components (Zhang and Calderwood, 2011). This is beneficial in clearing aggregated proteins and damaged organelles, reducing cellular stress and potentially slowing neurodegeneration (Cherra, 2008; Corti et al., 2020). Furthermore, HT can enhance blood circulation, improving the delivery of oxygen and nutrients to affected brain regions. This improved circulation can also facilitate the removal of metabolic wastes and toxins, which are often accumulated in NDs (Zagrean et al., 2017). This improved metabolic environment may support neuronal survival and function. Additionally, the thermal stress from HT may activate neuroprotective pathways, including the release of neurotrophic factors and modulation of inflammatory responses which both support control of chronic detrimental effects on neural tissue with increased neuron survival and regeneration. Research into NDs such as AD and/or HDs, which are characterized by the harmful build-up of amyloid peptides and misfolded tau proteins, has shown that exposing neuronal cells to mild HT between 42 and 45°C can lessen the toxicity caused by these proteins. This therapeutic effect is attributed to the activation of HSPs and the decreased activity of amyloid proteins in their phosphorylated forms (Behl and Schubert, 1993; Johnson et al., 1993). Additionally, Bastus et al. have shown that toxic amyloid peptides, specifically the Aβ1–42 sequence, can be eliminated by treating them with gold nanoparticles followed by microwave-induced heating, successfully breaking down the peptides (Kogan et al., 2006; Bastus et al., 2007). Regarding the behavior of linear amyloid fibrils (LAFs), one study observed the initial unfolding of these fibrils at temperatures above 51°C (Meersman and Heremans, 2003). Finally, Hu et al. recently described how HT induced by near-infrared laser-irradiated CsWO3 nanoparticles affects the disassembly of LAFs (Hu et al., 2020). Thus, the potential of the HT properties induced by these near-infrared laser-irradiated CsWO3 nanoparticles to break apart the fibrils, providing insight into new therapeutic strategies for tackling protein aggregation in NDs. Finally, Magnetic HT (MHT), a precision-targeted HT treatment, has shown considerable promise in the fight against aggressive brain tumors (Rivera et al., 2023). Through numerous clinical and preclinical trials, MHT has been explored not only as a standalone treatment but also as a complementary approach to existing therapies. Initial findings from these studies reveal MHT's potent antitumor capabilities in animal models and its positive impact on survival rates among human glioma patients (Paltanea et al., 2024). This emerging treatment method offers a glimpse into the future of brain cancer therapy, and beyond its immediate therapeutic potential, the application of MHT in treating brain tumors opens new avenues for understanding brain physiology and the mechanisms underlying NDs. By studying the effects of HT on brain tumors, researchers can gain invaluable insights into the brain's response to increased temperatures, including the activation of HSPs and the modulation of neuroinflammatory pathways (Dukay et al., 2019). This knowledge could pave the way for innovative HT-based treatments for NDs, offering hope for more effective management of diseases such as AD and PDs. Table 4 summarizes some of the studies exploring the effects of HT across different temperature ranges, durations, and application methods, highlighting its impact on HSPs expression, pathophysiological mechanisms, and therapeutic potential in both human and animal models of NDs.

**Table 4:** Comparative analysis of temperature-based applications in neurological disorders and their effects on heat shock proteins (HSPs) across human and animal models.

**References**	**Temperature Range (°C)**	**Time of application**	**Mode of application**	**Observed effect**	**Human or animal model**
Sharma and Hoopes (2003)	38–42	30–120 min	Whole-body hyperthermia	Pathophysiology of the CNS	Animal
Moseley (1997)	38–41	Repeated sessions over days	Environmental heating	Heat adaptation and HSP expression	Human
Horowitz and Robinson (2007)	40–43	Variable	Localized or systemic hyperthermia	HSP modulation	Human and Animal
Zulkifli et al. (2003)	40–42	Daily for a week	Environmental heat exposure	HSP70 expression and immune changes	Animal
Amorim et al. (2015)	–	Prolonged acclimation	Heat acclimation mechanisms	Increases intracellular Hsp72 levels	Human
Parsell et al. (1993)	41–43	Acute sessions	Localized heat application	Thermotolerance and HSP roles	Animal
Currie and White (1981)	39–41	Short-term	Thermal stress in tissues	Trauma-induced HSP roles	Animal
Currie and White (1983)	40	Short-duration exposure	Heat exposure	HSP synthesis characterization	Animal
Manzerra et al. (1997)	41	Short	Localized hyperthermia	HSP distribution in rabbits	Animal
Krueger et al. (1999)	42	Short-term	Localized heating	HSP70 expression in neurons	Animal
Bechtold et al. (2000)	42	Short-term	Localized heating	Synaptic HSP localization	Animal
Krueger-Naug et al. (2000)	42	Short-term	Localized heating	HSP27 in neuroglia and neurons	Animal
Bechtold and Brown (2003)	42	Short-term	Localized heating	HSP induction in hippocampal cells	Animal
Sasara et al. (2004)	42	Acute	Localized and systemic heating	HSP in spinal tissues	Animal
Tu et al. (2018)	40–42	Short-term	Intraperitoneal heat with chemotherapy	HSPs in gastric cancer	Human
Shetake et al. (2015)	43	Localized	Magnetic nanoparticle-mediated heat	Apoptosis via HSP modulation	Animal
Shetake et al. (2023)	40–42	Short	Localized hyperthermia	Therapeutic prognosis via serum HSP90	Human
Zhang and Calderwood (2011)	41	Acute	Localized heating	Autophagy and protein aggregation	Animal
Behl and Schubert (1993)	41	Short	Localized heating	Protection against amyloid-beta toxicity	Animal
Johnson et al. (1993)	41	Short	Localized heating	Phosphorylation of amyloid precursor	Animal
Bastus et al. (2007)	42	Short	Localized heat via nanoparticles	Amyloid disaggregation	Animal
Kogan et al. (2006)	–	Short	Nanoparticle-based hyperthermia	Protein aggregate manipulation	Animal
Meersman and Heremans (2003)	42	Short	Heat-induced dissociation	Denatured protein state	Animal
Hu et al. (2020)	42	Short	Laser-induced hyperthermia	Amyloid fibril disintegration	Animal
Rivera et al. (2023)	42	Variable	Magnetic hyperthermia	Neurosurgical tumor ablation	Human
Romeyke (2022)	40	no data available	Whole body hyperthermia	Post-COVID treatment	Human

## COVID-19 and Long COVID: a new health problem that could benefit from HT?

**Figure 3:**
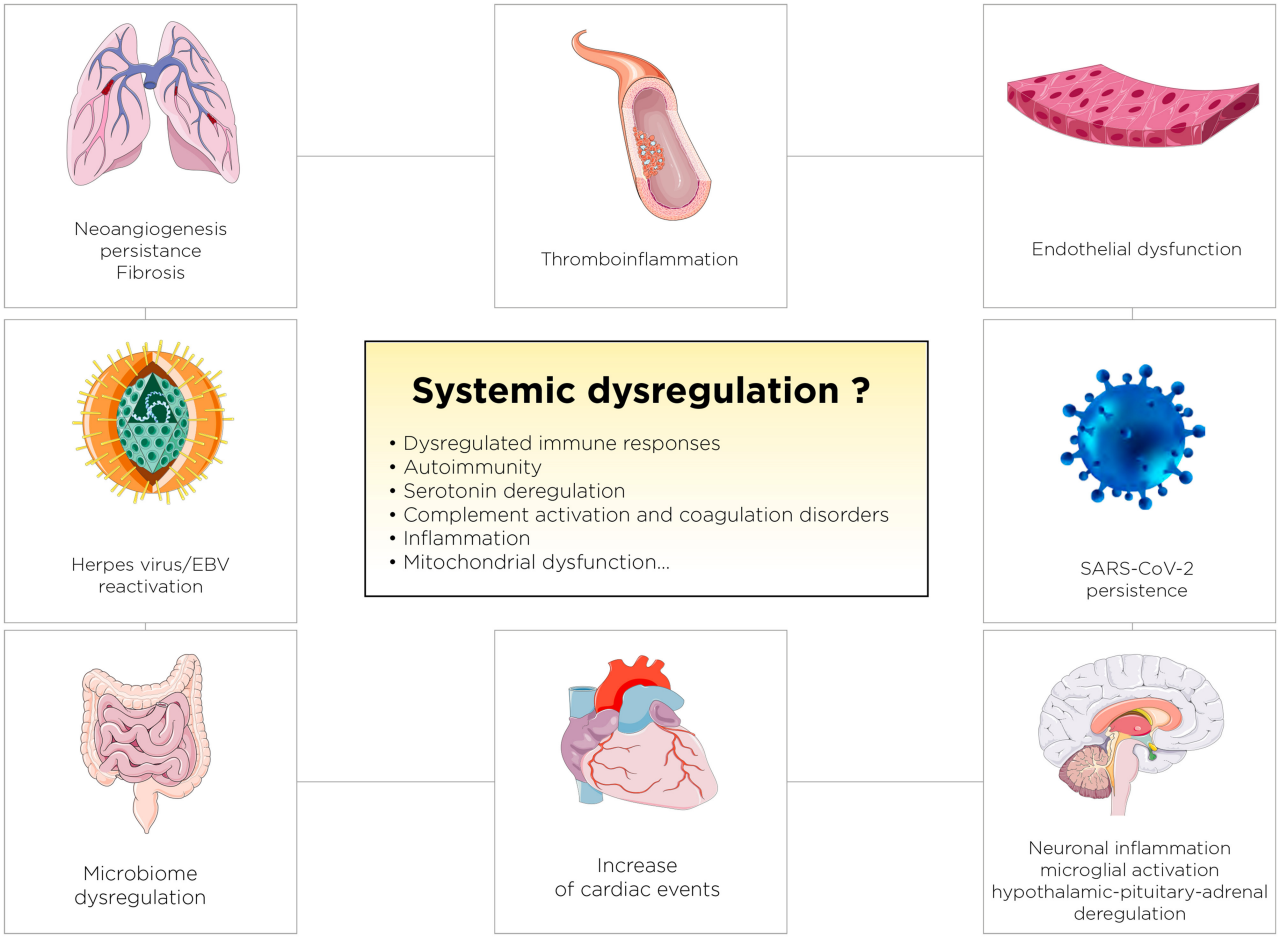
Proposed Pathophysiological Mechanisms Underlying Persistent Systemic Dysregulation in Long COVID. The figure illustrates key mechanisms, including endothelial dysfunction, thromboinflammation, neuroinflammation, immune dysregulation, and reactivation of latent viruses like EBV, contributing to long-term complications.

Long COVID represents a constellation of post-acute and chronic symptoms that vary widely across individuals, profoundly impacting public health and economies globally. Its prevalence has been estimated at around 6–30% of COVID-19 survivors, with manifestations ranging from fatigue and cognitive dysfunction to cardiovascular, neurological, and immune disorders. The global economic burden of Long COVID is staggering, with estimates of over $1 trillion annually due to healthcare costs, loss of productivity, and associated societal impacts (Antar and Cox, 2024). Long COVID, characterized by persistent symptoms following recovery from an acute SARS-CoV-2 infection, poses a significant challenge to healthcare systems worldwide (Montani et al., 2022; Davis et al., 2023). Symptoms range from fatigue and dyspnea to cognitive disturbances, often severely impacting quality of life. The exact mechanisms underpinning Long COVID remain unclear but are thought to involve persistent immune activation (Cervia-Hasler et al., 2024), endotheliopathy and coagulopathy (Fogarty et al., 2021), angiogenesis dysfunction (Philippe et al., 2023), and possibly viral reservoirs (Davis et al., 2023). Critically, there is no singular Long COVID; rather, it encompasses several subtypes, each likely governed by distinct pathophysiological mechanisms. This multifaceted nature underscores the urgency for personalized approaches to treatment and research. Figure 3 outlines key mechanisms, including viral persistence, immune dysregulation, mitochondrial dysfunction, and complement activation (Figure 3; Davis et al., 2023; Philippe et al., 2023; Al-Aly et al., 2024; Cai et al., 2024). Persistent viral reservoirs in immune-privileged sites can drive chronic inflammation, while dysregulated immune responses may trigger autoimmunity and latent viral reactivation. Additionally, mitochondrial dysfunction, characterized by impaired energy production and increased oxidative stress, contributes to the hallmark symptoms of fatigue and muscle weakness in Long COVID. HSPs, particularly HSP70 and HSP90, are also implicated as they play a dual role in protecting cells under stress while potentially perpetuating inflammation and apoptosis dysregulation. HSPs overexpression may disrupt mitochondrial function further, creating a feedback loop that exacerbates systemic and localized symptoms. A particularly debilitating subtype is neuro Long COVID, characterized by symptoms such as “brain fog,” memory loss, and difficulty concentrating (Narayanan et al., 2024; Slama Schwok and Henri, 2024). These symptoms are thought to stem from neuroinflammation, microglial activation, and impaired serotonin signaling. Evidence suggests that SARS-CoV-2 infection may lead to damage in the olfactory epithelium and brain regions, disrupting neuronal connectivity and triggering persistent inflammatory responses. Alterations in cerebrovascular function and immune activation within the central nervous system may further exacerbate cognitive deficits. Mitochondrial dysfunction and HSPs dysregulation in neurons could amplify these neurocognitive issues by impairing cellular energy metabolism and promoting prolonged stress responses in the brain. While strides have been made in identifying potential therapeutic targets, including interventions for serotonin depletion, HSPs modulation, and mitochondrial restoration, significant gaps in clinical evidence remain. Neuro Long COVID, in particular, highlights the importance of addressing brain health, energy metabolism, and cognitive rehabilitation in therapeutic strategies (Narayanan et al., 2024; Slama Schwok and Henri, 2024). Current knowledge emphasizes the need for continued investment in global surveillance, research, and therapeutic innovation to address the complex and pervasive challenge of Long COVID but also COVID-19.

First, the use of HT in acute COVID-19 management presents intriguing possibilities but requires careful consideration and rigorous validation. Ramirez et al. proposed hydrothermotherapy as a potential intervention to prevent or mitigate mild to moderate COVID-19 cases (Ramirez et al., 2021). Their hypothesis, based on the heat sensitivity of coronaviruses, suggests that artificial fever could enhance host immunity and antiviral responses, although no clinical data currently support this approach. Similarly, historical data from the Spanish flu, where heat treatments reduced mortality, lend indirect support to this hypothesis. However, as highlighted by Choron et al., fever and HT must be approached with caution, particularly in critically ill ICU patients (Choron et al., 2021). Their study identified HT above 103°F as a predictor of increased mortality, with outcomes worsening as temperatures rose further, underscoring the potential risks of uncontrolled temperature elevation. This aligns with findings by Rébé et al., who explored the role of HSP70 induced by controlled HT (Rébé et al., 2022). HSP70 can inhibit key inflammatory cytokines like TNF-α and IL-1β by targeting pathways such as NF-κB and inflammasomes, suggesting that heat could help mitigate the cytokine storm seen in severe COVID-19 cases. While HSP70 modulation appears promising for controlling hyperinflammation, it remains a hypothesis requiring further clinical exploration. Kasperkiewicz and Tukaj propose intriguing hypotheses regarding the role of HSPs, particularly HSP70 and HSP90, in the context of acute COVID-19 (Kasperkiewicz and Tukaj, 2022). Their research highlights the dual role of these proteins in both viral pathogenesis and potential therapeutic interventions. Moreover, HSP90 appears to facilitate the replication of SARS-CoV-2 by aiding in processes such as viral entry and propagation within host cells (Kasperkiewicz, 2021). This relationship is further complicated by molecular mimicry between SARS-CoV-2 antigens and human HSPs, particularly HSP60 and HSP90, which may contribute to autoimmune phenomena, including systemic disease manifestations like widespread microvascular damage. These findings suggest that HSP90 inhibitors, which have demonstrated efficacy in experimental models of autoimmune diseases, might also mitigate the cytokine storm and acute respiratory distress syndrome (ARDS) associated with severe COVID-19. Further elaborating on these ideas, Kasperkiewicz and Tukaj highlight the immunomodulatory effects of HSP70 and HSP90, which are overexpressed in response to stress and infection (Kasperkiewicz and Tukaj, 2022). While intracellular HSP70 generally exerts anti-inflammatory effects by suppressing NF-κB activation, extracellular HSP70 and HSP90 may exacerbate inflammatory processes and support viral replication. In COVID-19, the upregulation of these proteins appears to be a double-edged sword. On one hand, they could be leveraged to modulate immune responses and reduce the severity of the cytokine storm. On the other hand, their involvement in viral replication and autoimmune-like mechanisms warrants caution. The authors propose that targeted inhibition of HSP90, already under investigation for other conditions, could be a promising therapeutic approach to disrupt the interplay between SARS-CoV-2 and host cellular machinery, potentially curbing inflammation and viral proliferation. In summary, the hypotheses by Kasperkiewicz underscore the complex role of HSPs in acute COVID-19. While therapeutic targeting of these proteins, particularly HSP90, holds promise for reducing inflammation and viral activity, it remains critical to balance such interventions to avoid unintended consequences, such as exacerbating autoimmune responses. These hypotheses await robust clinical testing to validate their therapeutic potential. Thus, while HSPs targeting and HT-based interventions in COVID-19 offer potential therapeutic benefits, their implementation demands a balanced and evidence-based approach to maximize efficacy while avoiding harm.

## Serotonin, NDs, and Long-COVID

Serotonin, or 5-hydroxytryptamine (5-HT), is synthesized from the amino acid tryptophan (Modoux et al., 2021). Once produced, serotonin acts on various receptors throughout the body, categorized into seven main families (5-HT1 to 5-HT7), each with different subtypes and functions. Serotonin is a key neurotransmitter involved in mood regulation, sleep, and digestion, among other physiological processes. There are direct links between HSPs and serotonin (Kuwabara et al., 1994; Leja-Szpak et al., 2015). Indeed, serotonin can influence the stress response and modulate the expression of certain HSPs in the brain (Rensing and Monnerjahn, 1996; Yang and Lin, 1999; Bharti et al., 2015). For example, serotoninergic signaling has been implicated in the brain's response to heat stress, suggesting that serotonin might influence the expression of HSPs during such events (Tatum et al., 2015). Moreover, HSPs might protect serotoninergic neurons from stress-induced damage, thus maintaining serotonin levels and signaling (Ádori et al., 2006; El-Kasaby et al., 2014). For example, an imbalance between serotonin and HSP70 has been found in patients with Obsessive Compulsive Disorder (Cetin et al., 2023). Moreover, serotonin also plays a role in thermoregulation (Schwartz, 1995; Horseman et al., 2022). Serotoninergic pathways in the central nervous system can influence the body's response to changes in environmental temperature, contributing to mechanisms of heat loss or heat production (Morrison and Nakamura, 2019). For instance, serotonin may be involved in the behavioral and physiological responses to overheating, such as seeking shade or increasing blood flow to the skin (Bastus et al., 2007). Both HSPs and serotonin are involved in the brain's response to various stressors, including thermal stress (Sharma and Hoopes, 2003; Muchowski and Wacker, 2005; Horseman et al., 2022; Lukácsi et al., 2024). HSPs help protect neurons by preventing protein aggregation and aiding in the recovery of cells from heat-induced damage. Serotonin, through its diverse roles in the brain, can modulate the stress response, potentially affecting the expression of HSPs and the overall resilience of the brain to thermal and other forms of stress. Serotonin influences a wide range of brain functions through its extensive receptor family and neural pathways. Beyond the classic dopaminergic deficits in PD, serotonergic dysfunction has been identified, particularly affecting mood and gastrointestinal motility (Huang et al., 2023). Serotonin may also play a role in modulating motor functions and the progression of neurodegeneration in PD (Huot et al., 2011). Indeed, the disruption of the serotonergic system plays a crucial role in synucleinopathies such as PD, dementia with Lewy bodies (DLB), and Multiple System Atrophy (MSA; Halliday et al., 2014; Hsam and Kohl, 2023). Serotonin levels, in particular in platelet, have been found decreased in AD, potentially contributing to cognitive decline, mood disturbances, and sleep disruption commonly observed in patients (Tajeddinn et al., 2016a). Serotonergic system alterations may precede and contribute to the development of AD pathology, including β-amyloid accumulation and tau hyperphosphorylation (Rodríguez et al., 2012). There's compelling evidence linking serotonin neurotransmission to the formation of β-amyloid and tau protein aggregates (Rajmohan and Reddy, 2017). Involvement of various serotonin receptors have been demonstrated and the cellular signaling pathways they activate in the disease's progression, particularly in the aggregation of proteins, open new way of treatment in NDs (Rodríguez et al., 2012; Tajeddinn et al., 2016b). Alterations in the activity of specific serotonin receptors or their cellular signaling pathways may hinder the formation of β-amyloid plaques and tau protein neurofibrillary tangles (Sharma et al., 2021). Experimental findings suggest targeting serotonin receptors could not only enhance cognitive functions in AD patients but also play a crucial role in addressing the underlying causes of dementia associated with AD (Upton et al., 2008). Finally, in HD is associated with alterations in serotonin receptors, which may contribute to the psychiatric symptoms and cognitive decline observed in HD patients (Nithianantharajah and Hannan, 2013; Huang et al., 2023). In the context of thermoregulation, serotonin's effect is complex, involving multiple receptors that can either promote or inhibit heat production mechanisms (Voronova, 2021). Relationship between serotonin and heat have been largely described during serotonin syndrome (or serotonin toxicity; Krishnamoorthy et al., 2010; Chiew and Isbister, 2024). Serotonin-induced HT can occur through several mechanisms, notably involving the activation of the serotonin 2A receptor, which plays a pivotal role in thermoregulation (Nakamura et al., 2018). This receptor's stimulation can lead to increased heat production and reduced heat dissipation, culminating in HT (Nakamura et al., 2018). This phenomenon is a critical aspect of serotonin syndrome, a potentially life-threatening condition caused by excessive serotonergic activity. The relationship between HT and serotonin levels in the brain and blood is a fascinating area of research, focusing on how elevated body temperatures can impact serotonergic activity (Sharma et al., 1992; Hale et al., 2011, 2017, 2019). Studies suggest that HT can lead to an increased release of serotonin in the brain. This is thought to occur as a result of stress and heat-shock responses, which activate serotonergic neurons, leading to elevated serotonin release (Hale et al., 2011). HT may alter the sensitivity of serotonin receptors (Hale et al., 2011). Prolonged periods of elevated body temperature can lead to adaptations in receptor density and sensitivity, potentially as a protective mechanism against overstimulation by increased levels of serotonin (Sharma et al., 1992). The interaction between HT and serotonin levels underscores the importance of serotonin in thermoregulation.

In acute COVID-19, serotonin dysregulation has been directly implicated in documented cases of serotonin syndrome (SS) in two patients treated with lopinavir/ritonavir (LPV/r; Mas Serrano et al., 2020). SS, a life-threatening condition caused by excessive serotonin activity in the central and peripheral nervous systems, was likely triggered in these cases through pharmacological interactions involving LPV/r and other serotonergic medications (duloxetine, lithium, risperidone, and morphine). LPV/r exacerbated serotonergic activity by inhibiting key drug metabolism pathways (CYP3A4 and CYP2D6), amplifying serotonin levels. The authors highlight the classical clinical triad of SS—altered mental status, autonomic instability, and neuromuscular abnormalities—and emphasize the risks of co-prescribing serotonergic and antipsychotic drugs with LPV/r. These findings underline the importance of careful drug selection and dosing adjustments, especially in vulnerable populations, while raising broader questions about the role of serotonin in both acute and Long COVID.

**Figure 4:**
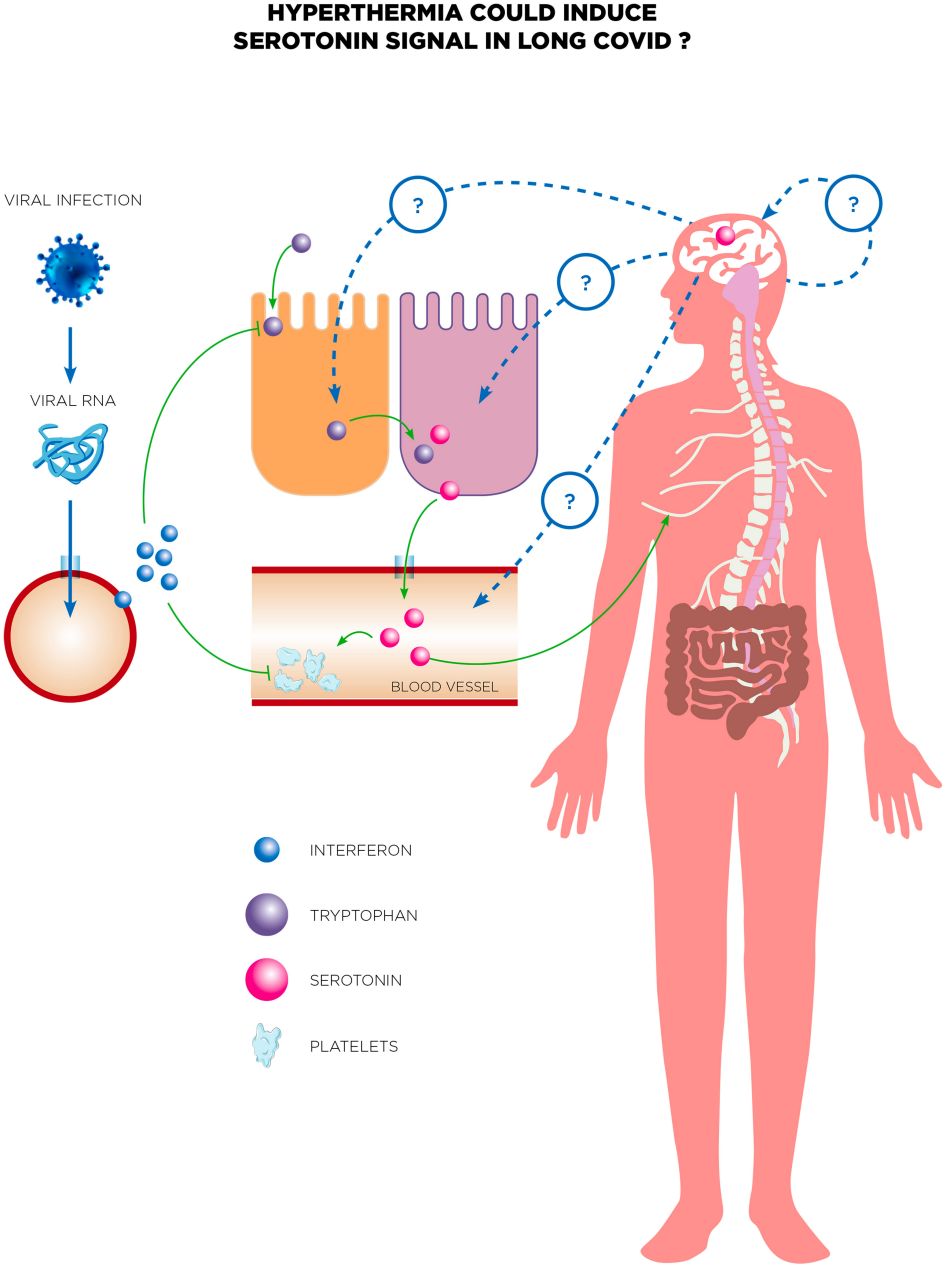
Serotonin is proposed as a potential biomarker for neurological decline and cognitive decline associated with long COVID. The reduction of peripheral serotonin driven by viral RNA negatively affects neurocognitive functions by weakening vagal signaling. In individuals suffering from long COVID, persistent viral RNA and resulting chronic inflammation led to decreased peripheral serotonin levels via the type-I IFN-IFNAR-STAT1 signaling pathway. Additionally, reduced tryptophan absorption, compromised serotonin storage, and increased serotonin breakdown contribute to this serotonin reduction. The systemic decrease in serotonin subsequently impairs neurocognitive abilities by directly weakening vagal signaling. Long COVID patients often experience neurological symptoms, highlighting the need for effective treatments. There are several potential therapeutic avenues for addressing these symptoms, including recent tests of whole-body HT treatment. This treatment has shown promising initial results in a case report of a long COVID patient experiencing neurological symptoms. While there is no current evidence linking serotonin to the efficacy of whole-body HT, this hypothesis warrants thorough exploration in future research to investigate the role of serotonin in the therapeutic efficacy of treatments for long COVID.

Furthermore, serotonin has also started to be a focus during Long COVID. Emerging evidence suggests that COVID-19 may disrupt serotonin levels and signaling pathways, contributing to the symptomatology of Long COVID. In a study by Wong et al., they conducted a metabolomic examination across groups of individuals experiencing acute COVID-19 symptoms, prolonged COVID symptoms, and those who recovered without symptoms (Wong et al., 2023). The analysis revealed a notable association between long-term COVID conditions and reduced serotonin levels. During the acute phase of COVID-19, serotonin levels dropped and failed to rebound in those suffering from Long COVID, whereas levels normalized in individuals who recovered fully. Additionally, artificially simulating a viral infection in mice through repeated doses of synthetic double-stranded RNA also led to decreased serotonin. A potential link has been proposed as a result of activating the type I interferon (IFN) pathway inducing reduced tryptophan levels, increased in platelet activation and a decrease in their numbers and also an increased activity of the monoamine oxidase (Mao) enzyme in the intestines indicating an uptick in serotonin metabolism amidst viral inflammation. The study also linked serotonin's vital role in cognitive functions to the observed serotonin deficit induced by the virus, which was associated with cognitive impairments in treated mice due to diminished sensory nerve activity and subsequent hippocampal response reductions (Figure 4). Remarkably, cognitive abilities in these mice were restored through treatments with selective serotonin reuptake inhibitors (SSRIs) or glycine-tryptophan dipeptide supplementation. Finally, serotonin could be a biomarker of NDs and cognitive decline associated to Long COVID and open the way toward potential therapeutic avenues for addressing neurological symptoms. For Long COVID, Eslami and Joshagami explored the relationship between serotonin levels and cognitive impairments in individuals suffering from Long COVID-19, highlighting how the immune response to SARS-CoV-2 impacts serotonin metabolism (Eslami and Joshaghani, 2024). It reveals that inflammation triggered by the virus impairs the absorption of dietary tryptophan, hinders serotonin transport by platelets, and increases enzymatic breakdown of serotonin, resulting in significantly lower serotonin levels in individuals with Long COVID compared to those who have fully recovered. This reduction in serotonin is linked to cognitive deficits such as difficulties in concentration and memory, as well as mood disorders like anxiety and depression. The study also identifies that the cytokine storm associated with acute COVID-19 exacerbates serotonin depletion, compounding these symptoms. Therapeutic strategies proposed include dietary supplementation with tryptophan and the use of selective serotonin reuptake inhibitors (SSRIs) such as fluoxetine, which have shown promise in improving memory and hippocampal activity in preliminary animal studies. The authors discuss the broader implications of immune system activation on neurotransmitter regulation, with particular attention to the kynurenine pathway, which diverts tryptophan from serotonin production during inflammation. These findings underscore the need for clinical trials to validate the efficacy of these interventions and to further investigate the complex interactions between the immune system and neurotransmitter pathways in Long COVID.

## The therapeutic potential of WBH: addressing NDs and Long COVID

Whole-body hyperthermia offers distinct advantages for NDs by leveraging systemic physiological responses such as improved mitochondrial function, HSPs induction, and modulation of neuroinflammation, which are not easily replicated by pharmacological treatments. Fever-range WBH (FRWBH), operating at 38.5–40.5°C, mimics natural fever states, enhancing both innate and adaptive immune responses while maintaining safety and patient comfort (Heckel-Reusser, 2022; Zschaeck and Beck, 2022). Advanced technologies, such as infrared-A irradiation (Müller et al., 2022; Piazena et al., 2022a,b), allow precise temperature control, making WBH feasible and potentially accessible with proper resource allocation. HSPs induction, particularly HSP70 and HSP90, is central to WBH's benefits, as these proteins help mitigate protein misfolding and aggregation, key factors in diseases like PDs (Ahn and Jeon, 2006). Moreover, WBH's ability to reduce oxidative stress and influence tau protein dynamics further supports its therapeutic relevance (Shepley et al., 2021). However, there is a critical need to understand its long-term impact on neuroinflammatory states, as HT could potentially exacerbate inflammation in predisposed patients. This underscores the importance of careful patient selection and stringent monitoring. While WBH shows promise as a complementary therapy, enhancing drug delivery and synergizing with neuroprotective agents, further clinical trials are essential to validate its safety and efficacy across diverse patient populations and inflammatory profiles. To seamlessly transition from a discussion on the relationship between serotonin and HT to the promising use of WBH for treating major depressive disorder (MDD), consider the following paragraph: the intricate relationship between serotonin and HT reveals the significant impact that body temperature regulation can have on neurotransmitter activity. Given serotonin's crucial role in mood regulation and its association with depression, therapies that can modulate serotonin levels are of used in clinic for some years. This understanding has paved the way for innovative treatments such as WBH. Indeed, WBH has emerged as a promising treatment for MDD. WBH is showing promise as a novel treatment for MDD. Janssen et al. conducted a randomized controlled trial that demonstrated WBH's specific antidepressant effects compared to a sham condition, highlighting its safety and efficacy (Janssen et al., 2016). A study recently explored the feasibility of combining WBH with cognitive behavioral therapy (CBT) in treating depression, finding significant reductions in depressive symptoms among participants (Mason et al., 2024). Another study by Mac Giollabhui et al. recently highlighted that the antidepressant effects of WBH are associated with the activation of the classical interleukin-6 signaling pathway, suggesting an immunomodulatory mechanism (Mac Giollabhui et al., 2024). Flux et al. further supported the antidepressant potential of WBH by showing that it could induce rapid and sustained improvements in mood and depressive symptoms, likely mediated through immune system interactions (Flux et al., 2023). Knobel et al. discussed the promising evidence of WBH in psychiatry, particularly its mood-enhancing effects and potential mechanisms involving immune modulation and serotonergic neurotransmission (Knobel et al., 2022). Hanusch et al. examined the associations between WBH and thermoregulatory cooling, further reinforcing WBH's potential as a treatment for depression (Hanusch et al., 2013). Hanusch and Janssen conducted a systematic review confirming WBH's impact on mood and depression, although they called for more evidence to make general clinical recommendations (Hanusch and Janssen, 2019). Collectively, these studies underscore WBH as a promising therapeutic modality for MDD, warranting further research to fully understand its mechanisms and optimize its clinical application (Knobel et al., 2022).

Whole-body hyperthermia has recently been explored as a treatment for a Long COVID patient suffering from neurological symptoms, yielding promising preliminary results (Romeyke, 2022). While the precise mechanisms behind its efficacy remain unclear, one intriguing hypothesis revolves around the potential role of serotonin in Long COVID (Figure 4). Could serotonin, a neurotransmitter already known for its impact on mood and inflammation, hold the key to understanding HT's impact in these cases? This bold idea beckons further investigation. Equally fascinating is the lack of data on HSPs in Long COVID, despite their established roles in cellular protection and immune modulation. To date, the solo case report has examined HT as a therapeutic option for Long COVID, leaving a vast frontier of research to uncover the interplay between HT, HSPs, and serotonin dysregulation in the persistence of symptoms. These emerging connections challenge us to rethink conventional approaches and open the door to groundbreaking discoveries in the treatment of Long COVID.

There are some potential clinical risks of HT in NDs and recent findings on how Long COVID uniquely affects neurological pathways could reinforce WBH's relevance. Firstly, specific clinical risks associated with HT in NDs or individuals with Long COVID must be carefully evaluated. In NDs, HT could exacerbate pre-existing vulnerabilities, such as impaired thermoregulatory capacity, autonomic dysfunction, or cardiovascular comorbidities often observed in these populations. Patients with compromised blood-brain barrier integrity may also experience heightened susceptibility to neuroinflammation or oxidative stress from elevated temperatures. Similarly, individuals with Long COVID, particularly those with post-viral autonomic dysfunction or chronic fatigue syndromes, might be at risk of worsening symptoms such as orthostatic intolerance, tachycardia, or systemic inflammatory activation. Thus, a targeted approach using WBH within carefully controlled temperature thresholds and under stringent monitoring is essential to mitigate these risks while harnessing its potential benefits. Secondly, recent findings on Long COVID's impact on neurological pathways strengthen the rationale for applying WBH. Long COVID is characterized by persistent neuroinflammation, microglial activation, mitochondrial dysfunction, and serotonin dysregulation. Emerging evidence indicates that SARS-CoV-2 may disrupt neurovascular integrity and neurotransmitter signaling, leading to symptoms such as “brain fog,” memory deficits, and chronic fatigue. WBH's ability to modulate inflammatory responses, enhance mitochondrial function, and induce HSPs could target these pathways, potentially ameliorating symptoms. By reducing oxidative stress, promoting protein homeostasis through HSPs, and improving cerebral perfusion, WBH may offer a unique and non-invasive therapeutic avenue for these patients. However, the variability of Long COVID symptoms necessitates individualized treatment plans and further clinical studies to validate WBH's efficacy.

Another point should be envisaged: indeed, potential pro-inflammatory risks and describe the importance of dose-controlled WBH to prevent adverse inflammatory responses. HT exerts a complex interplay between pro-inflammatory and anti-inflammatory effects, contributing to its therapeutic potential in modulating immune responses (Dieing et al., 2007; Repasky et al., 2013). On the pro-inflammatory side, HT enhances the release of cytokines such as IL-1 (Capitano et al., 2012), which play critical roles in promoting T-cell proliferation and immune activation (Atanackovic et al., 2002). These cytokines help recruit and activate key immune cells, including neutrophils, dendritic cells, and natural killer (NK) cells, amplifying their cytotoxic activity (Haveman et al., 1996). Additionally, HT stimulates the expression of HSPs, which act as danger signals to facilitate the cross-presentation of tumor antigens by antigen-presenting cells (APCs), thereby enhancing the activation of CD8+ cytotoxic T lymphocytes. This immune activation contributes significantly to anti-tumor responses by promoting a robust pro-inflammatory environment. Conversely, HT also induces anti-inflammatory effects that help regulate immune overactivation. It can increase the release of anti-inflammatory cytokines such as IL-10 (Zauner et al., 2014), while reducing levels of pro-inflammatory mediators like IL-12 and IFN-γ, particularly at higher or prolonged temperatures. This modulation reduces the risk of excessive inflammation and supports tissue recovery. HT also influences lymphocyte function and trafficking, promoting processes such as apoptosis in CD4+ T cells under stress conditions (Kobayashi et al., 2014). These mechanisms highlight HT's role in moderating immune responses to maintain a balance that prevents immune system overactivation while preserving its therapeutic benefits. This dynamic balance between pro-inflammatory and anti-inflammatory effects depends on factors such as temperature range, duration, and the specific context of HT application. Fever-range HT is particularly effective in stimulating immune responses without tipping into excessive suppression, making it a valuable adjunctive therapy in conditions such as cancer. Further research is needed to optimize HT protocols and tailor them to individual immune profiles, maximizing therapeutic efficacy while minimizing potential risks.

## Discussion, challenges, and conclusions

The current lack of clinical trial data specifically supporting WBH for NDs and Long COVID highlights the nascent stage of its exploration in these contexts. However, WBH's ability to induce HSPs, modulate neuroinflammation, and enhance mitochondrial function suggests its potential to address the key pathophysiological processes of these conditions. In NDs, these mechanisms may mitigate protein misfolding, reduce oxidative stress, and support neuronal survival, while in Long COVID, WBH's systemic effects could alleviate symptoms driven by persistent neuroinflammation and mitochondrial dysfunction. Advancing WBH as a therapeutic option requires clinical trials focused on safety, tolerability, and efficacy, particularly in populations with mild neurodegenerative symptoms or post-COVID cognitive impairment. These trials should aim to define optimal WBH parameters, such as temperature thresholds and treatment durations, to maximize therapeutic benefits while minimizing risks. Rigorous studies should also investigate WBH's effects on specific biomarkers, including inflammation and mitochondrial function, and evaluate its impact on clinical outcomes such as cognition, fatigue, and quality of life. Addressing challenges such as patient-specific risks and the accessibility of WBH technologies will require well-designed protocols and multidisciplinary collaboration. These steps will be essential for translating the promising mechanisms observed in preclinical studies into clinically actionable interventions.

All in all, exploring HT as a therapeutic strategy for NDs marks an exciting advancement in the field of medical science. By utilizing controlled heat and in particular brain thermal tunnel, there's the potential to alleviate the underlying pathological mechanisms of these diseases, offering new hope for reducing symptoms and decelerating their progression. The beneficial impact of HT, such as stimulating the production of HSPs and enhancing brain blood flow, highlights its therapeutic promise. Nonetheless, incorporating HT into established treatment regimens for NDs comes with its set of obstacles, including ensuring the safety of patients, discerning the long-term implications, and tailoring treatments to individual needs. Despite these challenges, current research efforts and the evolution of HT application technologies are laying the groundwork for future innovations. While HT introduces a novel and intriguing method for managing NDs, thorough research and clinical substantiation remain essential. Looking ahead, the successful adoption of HT in the management of these conditions could profoundly improve patient quality of life, emphasizing the critical role of ongoing exploration and development in this arena.
